# The Necessity of CT Hip Scans in the Investigation of Occult Hip Fractures and Their Effect on Patient Management

**DOI:** 10.1155/2021/8118147

**Published:** 2021-11-18

**Authors:** Thomas Gatt, Daniel Cutajar, Lara Borg, Ryan Giordmaina

**Affiliations:** Department of Orthopaedics and Trauma, Mater Dei Hospital, Msida MSD2090, Malta

## Abstract

The diagnostic challenge of negative plain radiography in the context of a previously ambulatory patient is increasing with the rise in geriatric trauma. These patients are often diagnosed with small undisplaced fractures of the pelvis and femur which may not alter management. This study aims to assess the frequency at which computed tomography (CT) hip scans altered patient management and whether two X-ray projections of the hip affected fracture detection rate. All CT hip scans performed over a three-year period were identified retrospectively. Only CT hips pertaining to the identification of occult fractures were included in the study. A total of 447 (63.6%) CT hips were performed to exclude an occult fracture, which was only detected in 108 (24.1%) of the scans requested. The majority were subcapital (*n* = 58, 53.7%) or intertrochanteric (*n* = 39, 36.1%). There was no significant difference between fracture detection rates when comparing one and two views of the pelvis. 82.4% (*n* = 89) of occult hip fractures were managed operatively. CT imaging led to a change in patient management in 20% of cases. The frequency at which CT scan detects and alters management in occult hip fractures confirms the justification for its use. Increasing the number of X-ray projection views does not decrease the reliance on CT. Pelvic ring fractures are common in nonambulatory patients following trauma, and if confirmed on initial imaging, subsequent imaging to exclude a concurrent occult hip is unnecessary. The focus of further research should be towards the development of investigation algorithms which decrease the reliance on CT and defining the optimal surgical criteria for occult hip fractures.

## 1. Introduction

Occult hip fractures are defined as fractures which are not visible on initial two-view conventional radiography of the hip. The incidence is reported to be as high as 10% on initial imaging [1]. Delayed diagnosis may lead to complications such as fracture displacement, delayed surgery, nonunion, and increased morbidity [2]. While magnetic resonance imaging (MRI) is the recognised gold standard for diagnosis, this is not always an easily accessible or cost-effective option in many centres. For this reason, there has been a move towards the use of computed tomography (CT) as first-line imaging for occult hip fractures, in view of its high sensitivity and specificity rates [3].

However, CT scanning is not without its own disadvantages. Even when using a lower dose protocol, CT is costly and leads to more radiation exposure than conventional radiography. Additionally, patients are subject to longer waiting times in the emergency department when subsequent imaging is requested following normal or inconclusive imaging. With respect to hip pathology in the acute setting, patients are often subjected to a single anteroposterior (AP) view of the pelvis, with some literature claiming that this is sufficient for fracture detection [4]. The diagnostic challenge of negative plain radiography in the context of a previously ambulatory patient is increasing with the rise in geriatric trauma. Often these patients are subsequently diagnosed with small undisplaced fractures of the pelvis and femur which might not alter management. This raises the question whether or not better initial imaging of the pelvis and hips can detect more subtle pathology, explaining the patient's symptomatology and decreasing reliance on CT.

This study aims to assess the frequency at which CT scans of the hip were deemed to change patient management. The secondary aims were to assess whether two projections of the hip had any effect on fracture detection rate and to obtain a better understanding of injury patterns in this patient cohort.

## 2. Method

All CT hip scans performed at Mater Dei Hospital, Malta, over a three-year period from January 1, 2018, to December 31, 2020, were identified retrospectively through the Centricity™ Universal Viewer Zero Footprint software. All CT hips pertaining to the identification of occult fractures were included in the study. Scans performed for chronic pain, preoperative planning, or postoperative complications were excluded. In our hospital, while there is currently no formal algorithm pathway for suspected occult hip fractures, CT is the commonly accepted imaging modality. Indications for CT imaging despite negative radiography included inability to weight bear, acute limb shortening following trauma, and severe pain on passive movement. Any patient who showed one of these indications formed part of the inclusion criteria to scan. Exclusion criteria were confirmed or suspected pregnancy and patient refusal. The decision to perform a CT hip is normally a joint decision between the emergency physician and the orthopaedic specialist on call, after reviewing the initial X-rays. On occasion, it may also be a recommendation in the X-ray report issued by the radiologist. Requests for CT scans need to be vetted by the CT radiologist on call.

For each scan performed, data on patient demographics, mechanism of injury, indication, time from injury, X-ray number of projections, X-ray findings, fracture types detected, and management outcomes were collected. Only the official imaging reports as issued by the medical imaging department were considered.

SPSS Statistics software was used to collect and analyse the data. Chi square testing was used to assess the relationship between categorical variables. A *p* value of <0.05 was deemed statistically significant.

## 3. Results

A total of 702 CT hip scans were performed over the study period. The majority of these were to exclude occult hip fractures (*n* = 447, 63.6%), with the rest performed for preoperative planning (*n* = 53, 7.5%), postoperative assessment (*n* = 71, 10.1%), and chronic conditions (*n* = 131, 18.7%) (Table 1).

Of the 447 suspected occult hip fractures analysed further, the majority were females (*n* = 256, 57.3%) with a mean age of 75.6 years (SD = 14.4). A fracture was detected in 251 cases (56.1%); however, an occult fracture of the proximal hip was only detected in 108 (24.1%) of the CTs requested. The other fractures were primarily isolated pubic ramus fractures (*n* = 51, 20.3%), isolated greater trochanter fracture (*n* = 31, 12.4%), isolated acetabulum fractures (*n* = 18, 7.2%), or a combination of pelvic ring fractures (*n* = 33, 13.1%). Of the 108 occult hip fractures identified, the majority were subcapital (*n* = 58, 53.7%) or intertrochanteric (*n* = 39, 36.1%) (Table 2).

An X-ray was taken prior to CT in the majority of cases (*n* = 432, 96.6%). X-rays performed were either a single AP view of the pelvis including both hips (*n* = 112, 25.9%), a single AP of the affected hip (*n* = 84, 19.4%), or an AP of the pelvis with a lateral view of the affected hip (*n* = 236, 54.6%). There was no significant difference between the proximal femur fracture detection rate when comparing AP view of the whole pelvis and AP view of only the affected hip (*p*=0.65). There was also no significant difference in fracture detection rate when comparing one-view and two-view radiography (*p*=0.22).

Of the 108 occult hip fractures, 82.4% (*n* = 89) were managed operatively. Operative fixation was performed most frequently by dynamic hip screw (*n* = 34, 31.5%), hemiarthroplasty (*n* =  28, 25.9%), or cannulated screws (*n* = 15, 13.9%) (Table 3).

CT imaging for suspected occult hip fractures led to a change in patient management in 20% of cases. Repeat X-ray imaging within 6 months, despite negative CT findings, was performed in 6 cases. In one instance, an old subcapital fracture was picked up, which was not visible on CT imaging. This patient underwent a total hip replacement, with good recovery.

## 4. Discussion

Previously, mobile patients who are unable to ambulate following trauma are a diagnostic challenge. The complications of overinvestigation must be balanced against the high morbidity of missing a hip fracture [5]. CT imaging is typically reserved for patients with a clinical suspicion of a hip fracture despite initial negative or inconclusive imaging. This includes, for instance, patients complaining of pain out of proportion to X-ray findings, pain on axial compression of the limb, or being unable to mobilize adequately following trauma. Unfortunately, these presentations are very common in frail individuals, and clinical signs cannot be relied upon to exclude an occult fracture [6]. While some authors have tried to describe tests to aid clinical diagnosis, such as the patellar percussion test, these are no replacement for imaging [7]. CT imaging has emerged as a cost-effective imaging alternative with very high sensitivity and specificity rates [1, 2, 8, 9]. The concern is that this may be used excessively and pick up clinically irrelevant pathology in this patient cohort, ultimately not altering the management or patient disposition significantly.

Our study confirmed similar findings from previous literature that the lateral view of the hip contributes little to detecting occult fractures and decreasing the subsequent need of CT imaging [4, 10]. However, the authors' experience is that it is still relevant in the detection of nonoccult hip fractures and planning surgical techniques. We therefore still recommend both AP and lateral radiographs of the hip as the initial imaging. Some studies have looked at performing novel Bristol views of the hip, which involves a 30-degree angulation film instead of a conventional lateral. If needed, it is found to be more comfortable for the patient, and initial studies show that it is superior to lateral views, despite not having been assessed in large cohorts yet [11].

A significant portion of the patients were found to have some degree of pelvic ring injury, either isolated or in combination. Our study confirmed that pelvic ring injuries and occult hip fractures tend not to occur simultaneously [12]. This also confirms that an established pelvic ring fracture does not always warrant further investigation for occult hip fractures [13]. Only 3 patients sustaining pelvic ring fractures required surgical fixation, and this was typically reserved for younger patients. Older patients are typically treated with a period of rest, gradual weight-bearing, and physiotherapy. Therefore, the CT scan did little to alter the management in these cases once a pelvic ring fracture was already identified [14].

In this study, the CT detection rate for occult fractures of 24.2% was similar to other studies, ranging from 13 to 39% [1, 2, 15, 16]. Greater trochanter (GT) fractures were mostly isolated, and only a minority extended to the capsule. This was in keeping with the findings of some reports [1]; however, it is in contrast to the findings from other studies which found that up to 90% are extending into the intertrochanteric region [16, 17]. In fact, Kim et al. recommend MRI for all isolated GT fractures, in view of it leading to significant fixation rates [18]. Operative fixation in our study was 82.4% and was significantly higher for occult hip fractures when compared to the meta-analysis performed by Haj-Mirzaian et al. [16] Conservative management for these fractures is generally reserved for older, medically unfit patients with incomplete fracture extending to less than 50% [19]. This discrepancy is probably due to variations in local practice; however, further study focusing on this discrepancy in fixation rates is warranted.

The fact that CT imaging changed the management in 20% of patients in this study shows that low-dose CT remains a relevant investigation for occult hip fractures. This would also be true if we were to have followed a more conservative fixation rate than this study recorded. It may also be argued that the remaining 80% of scans were needed to achieve a diagnosis and reassure patients. Some authors have attempted to devise an algorithm to guide management on initial presentation of this patient cohort. Such algorithms would ensure more stringent criteria to decrease unnecessary imaging. Gangopadhyay et al. proposed sending home ambulatory patients to undergo repeat X-ray in one week, or admitting nonambulatory patients to scan the following day if symptoms persist [20]. This may lead to reducing unnecessary CT scans in these patients. The development of such algorithms coupled with cost-effectiveness studies for these approaches should serve as the focus for further studies on the topic.

### 4.1. Limitations

This study is limited by its retrospective nature. Data were collected from the official X-ray and CT reports issued online by the radiology department. The medical grade of the person reporting is not documented, and this in itself may affect the data. It may well be that a more experienced radiologist may have picked up the more subtle signs, reducing the need for a CT scan, giving rise to a degree of sampling bias. Furthermore, the patient outcomes following the management of these occult fractures were unable to be obtained.

## 5. Conclusion

The frequency at which CT scan detects and alters management in occult hip fractures confirms the justification for its use. Increasing the number of X-ray projection views does little to increase detection rate and decrease reliance on CT. However, we still advocate a minimum of two views for the benefit of nonoccult hip fractures. Pelvic ring fractures are very common in nonambulatory patients following trauma, and if confirmed to be stable on initial imaging, subsequent imaging to exclude a concurrent occult hip is unnecessary. The focus of further research should be towards the development of investigation algorithms which decrease the need for CT and defining the optimal surgical criteria for occult hip fractures.

## Figures and Tables

**Table 1:** Indications for CT hip scans.

	*N* (%)
Total CT hip scans performed	702 (100)
Suspected occult hip fracture	447 (63.7)
Preoperative planning	53 (7.5)
Postoperative assessment	71 (10.1)
Chronic pathology	131 (18.7)
Longstanding hip pain	96 (13.7)
Malignancy	14 (2)
Infection	9 (1.3)
Postreduction	7 (1)
Miscellaneous	5 (0.7)

**Table 2:** Fracture patterns for positive CT hip results.

	*N* (%)
Fracture detected	
Yes	251 (56.2)
No	196 (43.8)
Fracture types	
Occult proximal femur	108 (43)
Pubic ramus	51 (20.3)
Greater trochanter	31 (12.4)
Acetabulum	18 (7.2)
Iliac crest	4 (1.6)
Sacrum	1 (0.4)
Combination^*∗*^	33 (13.1)
Others^#^	5 (2)
Occult proximal femur types	
Subcapital	58 (53.4)
Intertrochanteric	39 (36.1)
Basicervical	4 (3.7)
Transcervical	4 (3.7)
Pertrochanteric	1 (0.9)
Subtrochanteric	2 (1.9)

^*∗*^Combination patterns include any two or more of the following: ramus, sacrum, acetabulum, ilium, and ischium. ^#^Other fracture patterns include distal femur (1), femoral head (2), femoral shaft (1), and lesser trochanter (1).

**Table 3:** Outcomes of confirmed occult hip fractures.

	*N* (%)
Conservative	19 (17.6)
Dynamic hip screw	34 (31.5)
Hemiarthroplasty	28 (25.9)
Cannulated screws	15 (13.9)
Total hip arthroplasty	6 (5.5)
Proximal femoral nail	5 (4.6)
Open reduction internal fixation	1 (0.9)

## Data Availability

The data that support the findings of this study are available from the corresponding author upon reasonable request.
